# Supplementary material for: Robust and durable serological response following pediatric SARS-CoV-2 infection
Source: Nat Commun. 2022 Jan 10;13:128. doi: 10.1038/s41467-021-27595-9 (PMC8748910; doi:10.1038/s41467-021-27595-9)
Supplement: Supplementary file 1 — Supplementary Information [file 41467_2021_27595_MOESM1_ESM.pdf]

Supplementary Information – Robust and durable serological response following pediatric SARS-CoV-2 infection

Hanna Renk MD[1*], Alex Dulovic Dr.rer.nat[2*], Alina Seidel M.Sc[3*], Matthias Becker M.Sc[2], Dorit Fabricius MD[4], Maria Zernickel M.Sc[4], Daniel Junker M.Sc[2], Rüdiger Groß M.Sc[3], Janis A. Müller Dr.rer.nat[4], Alexander Hilger M.Sc[5], Sebastian F.N. Bode MD[4], Linus Fritsch[5], Pauline Frieh[5], Anneke Haddad DPhil[5], Tessa Görne[5], Jonathan Remppis MD[1], Tina Ganzemueller MD[7], Andrea Dietz Dr.biol.hum[6], Daniela Huzly MD[8], Hartmut Hengel MD[8], Klaus Kaier PhD[9], Susanne Weber Dipl.Math[9], Eva-Maria Jacobsen Dr.rer.physiol[4], Philipp D. Kaiser Dr.rer.nat[2], Bjoern Traenkle Dr.rer.nat[2], Ulrich Rothbauer Dr.rer.nat[2], Maximilian Stich MD[10], Burkhard Tönshoff MD[10], Georg F. Hoffmann MD[10], Barbara Müller PhD[11], Carolin Ludwig[12,13,14], Bernd Jahrsdörfer MD[12,13,14], Hubert Schrezenmeier MD[12,13,14], Andreas Peter MD[15], Sebastian Hörber MD[15], Thomas Iftner PhD[7], Jan Münch PhD[3], Thomas Stamminger MD[6], Hans-Jürgen Groß MD[16], Martin Wolkewitz PhD[9], Corinna Engel Dr.biol.hum[1,17], Marta Rizzi MD[18], Weimin Liu MD[19], Beatrice H. Hahn MD[19], Philipp Henneke MD[5,20], Axel R. Franz MD[1,17], Klaus-Michael Debatin MD[4], Nicole Schneiderhan-Marra Dr.rer.nat[2], Ales Janda MD[4,#] and Roland Elling MD[5,20,#,†]

1 – University Children's Hospital Tübingen, Tübingen, Germany

2 – NMI Natural and Medical Sciences Institute at the University of Tübingen, Reutlingen, Germany

3 – Institute of Molecular Virology, Ulm University Medical Center, Ulm University, Ulm, Germany

4 – Department of Pediatrics and Adolescent Medicine, Ulm University Medical Center, Ulm University, Ulm, Germany

5 – Center for Pediatrics and Adolescent Medicine, Medical Center Freiburg, Germany and Faculty of Medicine, University of Freiburg, Freiburg, Germany

6 – Institute of Virology, Ulm University Medical Center, Ulm, Germany

7 – Institute for Medical Virology and Epidemiology of Viral Diseases, University Hospital Tübingen, Tübingen, Germany

8 – Institute of Virology, Medical Center Freiburg, Germany and Faculty of Medicine, University of Freiburg, Freiburg, Germany

9 – Institute of Medical Biometry and Statistics, Medical Center Freiburg, Germany and Faculty of Medicine, University of Freiburg, Freiburg, Germany

10 – Department of Pediatrics I, University Children's Hospital Heidelberg, Heidelberg, Germany

11 - Department of Infectious Diseases, Virology, Heidelberg University Hospital, Heidelberg, Germany

12 – Department of Transfusion Medicine, Ulm University, Ulm, Germany

13 – Institute for Clinical Transfusion Medicine and Immunogenetics, Ulm, Germany

14– German Red Cross Blood Transfusion Service, Baden-Württemberg-Hessen, Germany

15 – Institute for Clinical Chemistry and Pathobiochemistry, University Hospital Tübingen, Tübingen, Germany

16 – Institute of Clinical Chemistry, Ulm University, Ulm, Germany

17 – Center for Pediatric Clinical Studies, University Hospital Tübingen, Tübingen, Germany

18 - Department of Rheumatology and Clinical Immunology, Medical Center Freiburg, Germany and Faculty of Medicine, University of Freiburg, Freiburg, Germany

19 - Department of Microbiology and Department of Medicine, University of Pennsylvania, Philadelphia, USA

20 – Institute for Immunodeficiency, Medical Center Freiburg, Germany and Faculty of Medicine, University of Freiburg, Freiburg, Germany

*These authors contributed equally

#These authors jointly supervised this work

†indicates corresponding author

Supplementary Figures

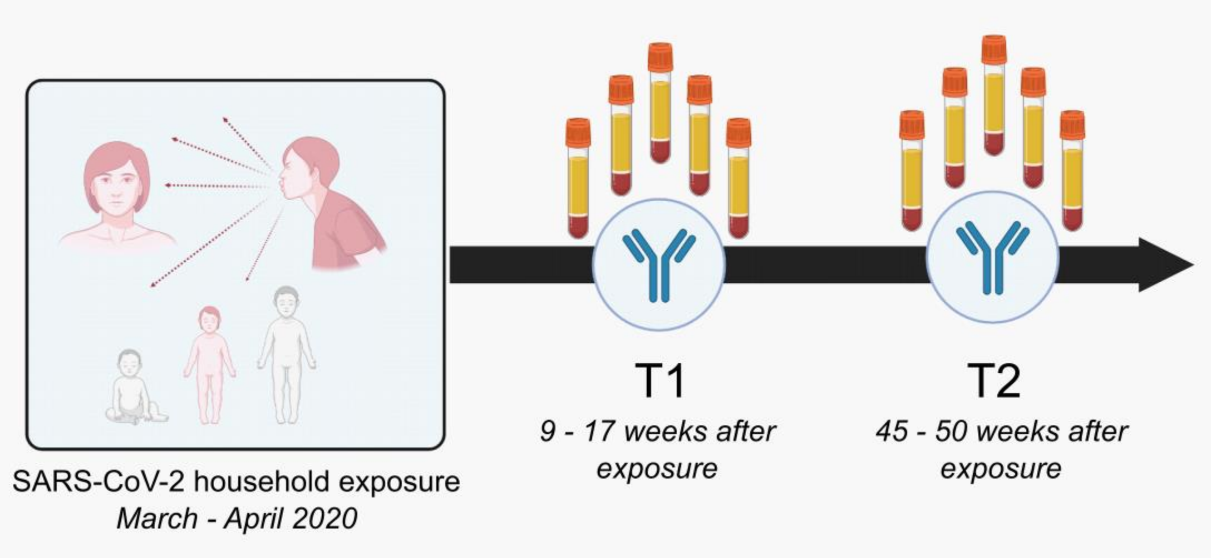

Figure S1: **Overview of the time points within the study population.** Illustration of study design, from exposure to study participation time points. Times shown are the IQR for each time point. T1 – Time point 1, T2 – Time point 2.

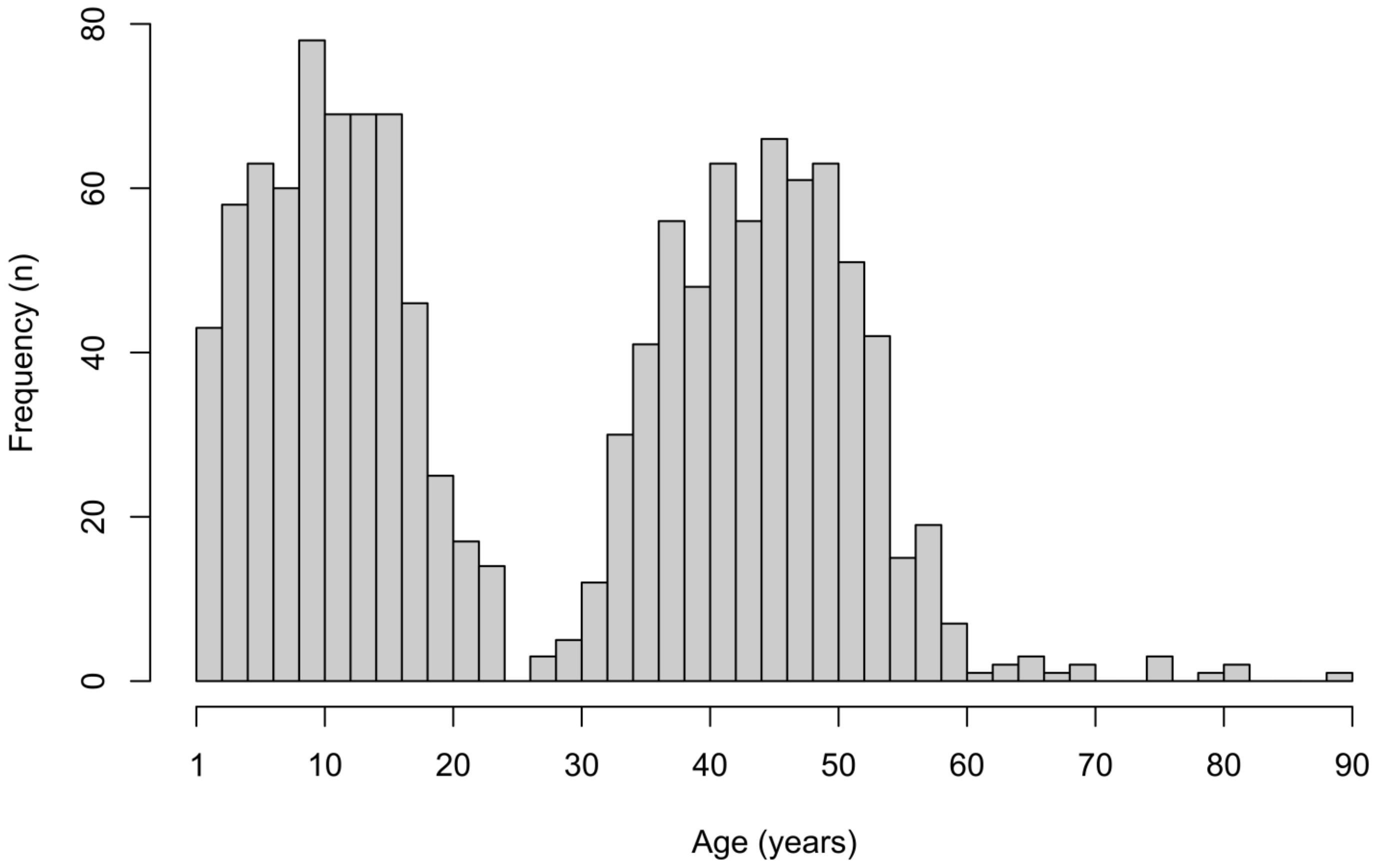

Figure S2 – **Study population age distribution**. Histogram showing age distribution within the study population at T1 (n=1265).

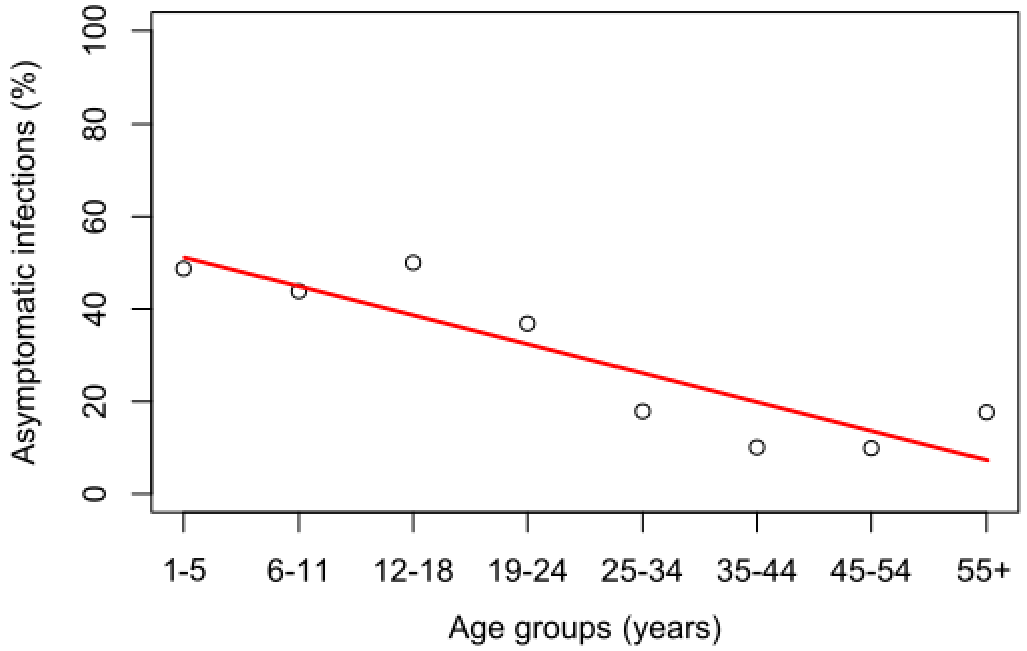

Figure S3 – **Proportion of asymptomatic infections decreases with age**. Line graph demonstrating the decrease in the proportion of asymptomatic infections with increasing age across age-group. Red line indicates line of best fit. Only samples at T1 were included in this analysis (n=1265).

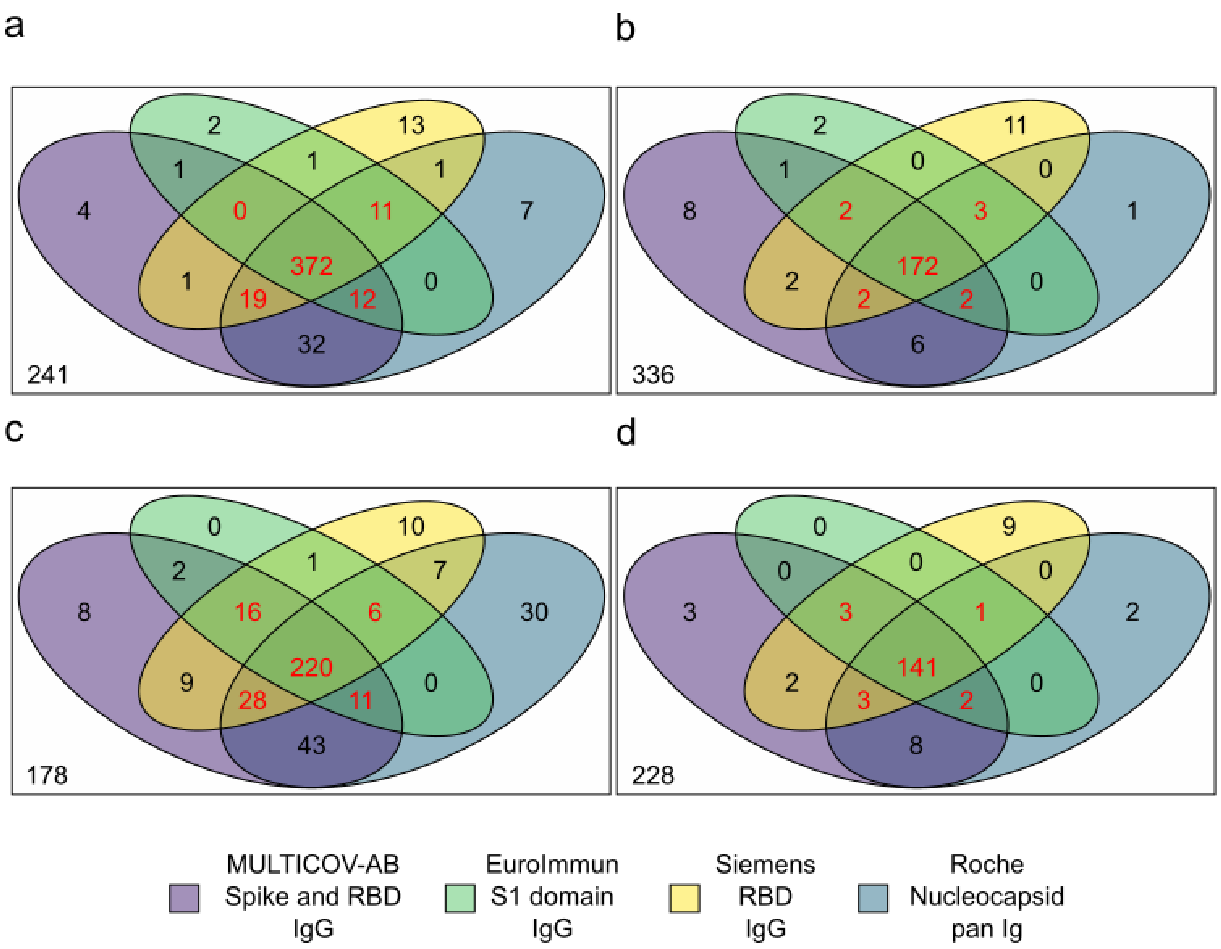

Figure S4 – **Comparative performance of the different serology assays used in this study**. 4-way Venn diagrams showing how each assay classified samples as being seropositive for T1 (a and b) and T2 (c and d) for adults (a and c) and children (b and d). Samples were classified as being positive if three or more assays classified them as being positive (shown in red). Negative samples for all assays are indicated in the bottom left corner of the boxes. Assays are color-coded as defined by the key including the manufacturer or name of the assay, which antigen it uses as a target and which Ig-isotype it measures. RBD – receptor binding domain.

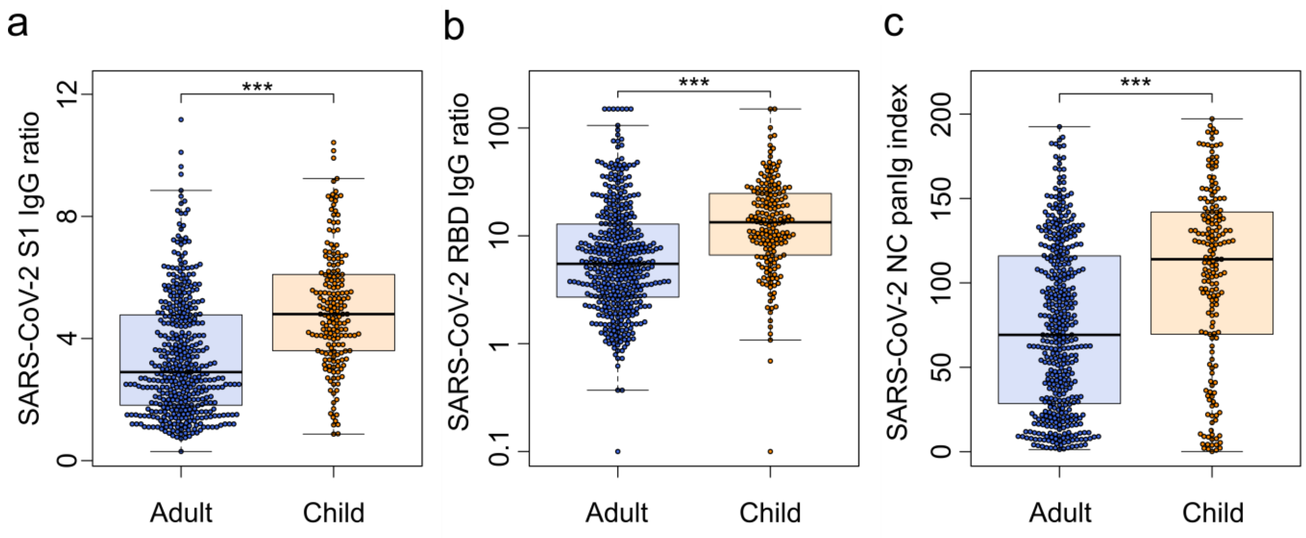

Figure S5 – **Children have higher antibody responses than adults**. Seropositive children (orange, n=181) had significantly higher IgG antibody titres against S1 (a, $p=1.52x10^{-18}$), receptor binding domain (RBD) (b, $p=7.05x10^{-14}$) and nucleocapsid (NC) (c, $p=7.55x10^{-10}$) than seropositive adults (blue, n=414) as determined using the commercial EuroImmun (a), Siemens (b) and Roche (c) assays at T1. The Ig isotype measured with each assay is indicated on the axis. Seropositive adults and children were identified using the multi-assay definition of seropositivity explained in the Method section. Box and whisker plots with the box representing the median, 25th and 75th percentiles, while whiskers show the largest and smallest non-outlier values. Outliers were identified using upper/lower quartile ± 1·5 times IQR. Statistical significance was calculated by Mann-Whitney-U (two-sided) with *** indicating a p-value <0·001.

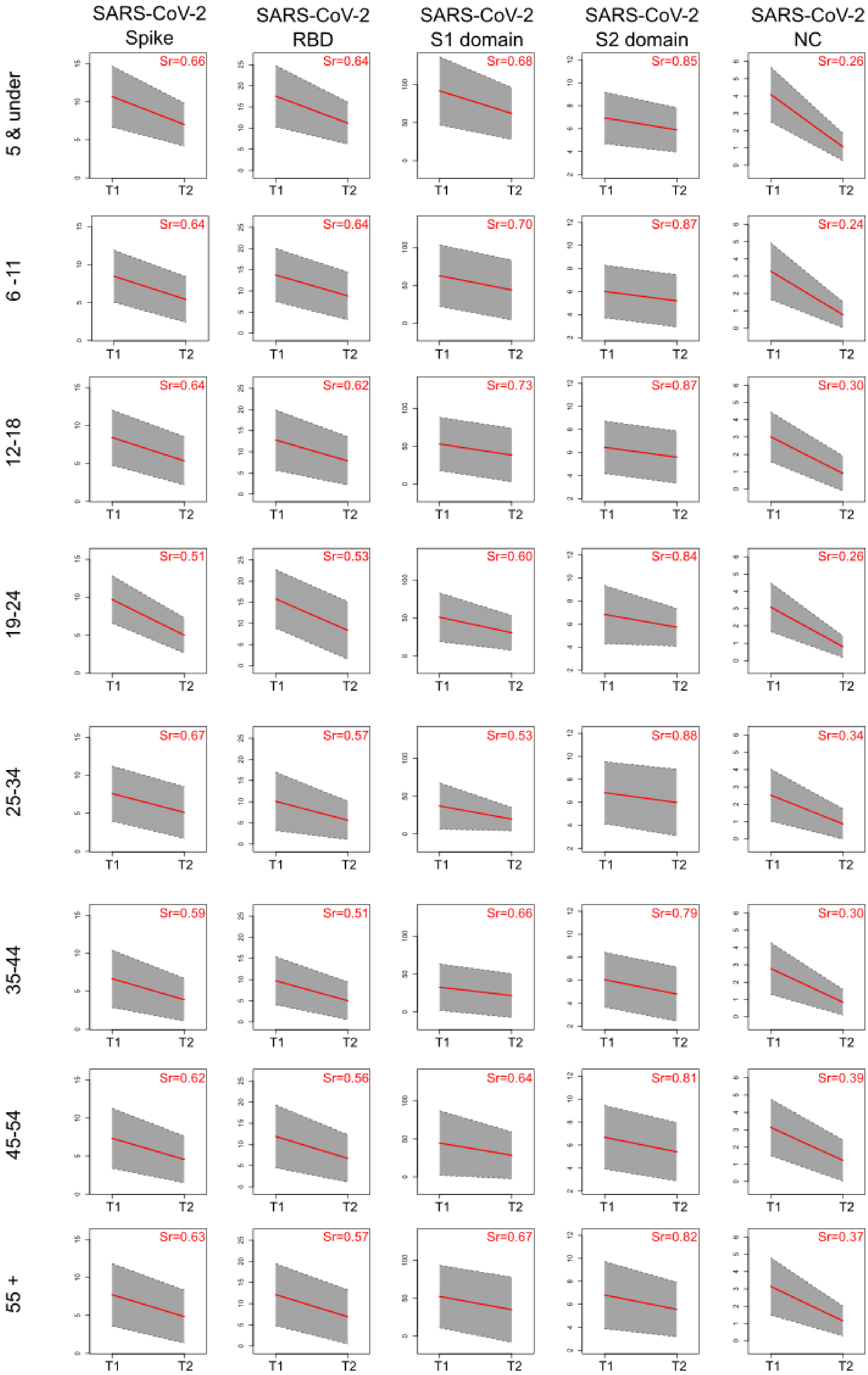
SARS-CoV-2 Spike
SARS-CoV-2 RBD
SARS-CoV-2 S1 domain
SARS-CoV-2 S2 domain
SARS-CoV-2 NC
5 & under
Sr=0.66
Sr=0.64
Sr=0.68
Sr=0.85
Sr=0.26
6 -11
Sr=0.64
Sr=0.64
Sr=0.70
Sr=0.87
Sr=0.24
12-18
Sr=0.64
Sr=0.62
Sr=0.73
Sr=0.87
Sr=0.30
19-24
Sr=0.51
Sr=0.53
Sr=0.60
Sr=0.84
Sr=0.26
25-34
Sr=0.67
Sr=0.57
Sr=0.53
Sr=0.88
Sr=0.34
35-44
Sr=0.59
Sr=0.51
Sr=0.66
Sr=0.79
Sr=0.30
45-54
Sr=0.62
Sr=0.56
Sr=0.64
Sr=0.81
Sr=0.39
55 +
Sr=0.63
Sr=0.57
Sr=0.67
Sr=0.82
Sr=0.37
T1
T2

Figure S6 – **Antibody decay occurs at the same rate in adults and children.** Longitudinal comparison of T1 and T2 samples using MULTICOV-AB to determine the rate of antibody decay. The IgG antibodies against spike trimer, receptor binding domain (RBD), S1 domain, S2 domain and nucleocapsid (NC) of SARS-CoV-2 are shown. Samples are separated into distinct age groups: under 5 years old (n=28), 6-11 (n=61), 12-18 (n=68), 19-24 (n=14), 25-34 (n=21), 35-44 (n=117), 45-54 (n=148) and over 55 years old (n=31). All y-axis show the normalized MFI. Red lines indicate mean rates of decrease; grey boxes indicate ±1 standard deviation. Sr indicates proportion of signal remaining, calculated as the ratio of the mean MFI at T2 compared to the mean MFI at T1. MFI – median fluorescence intensity.

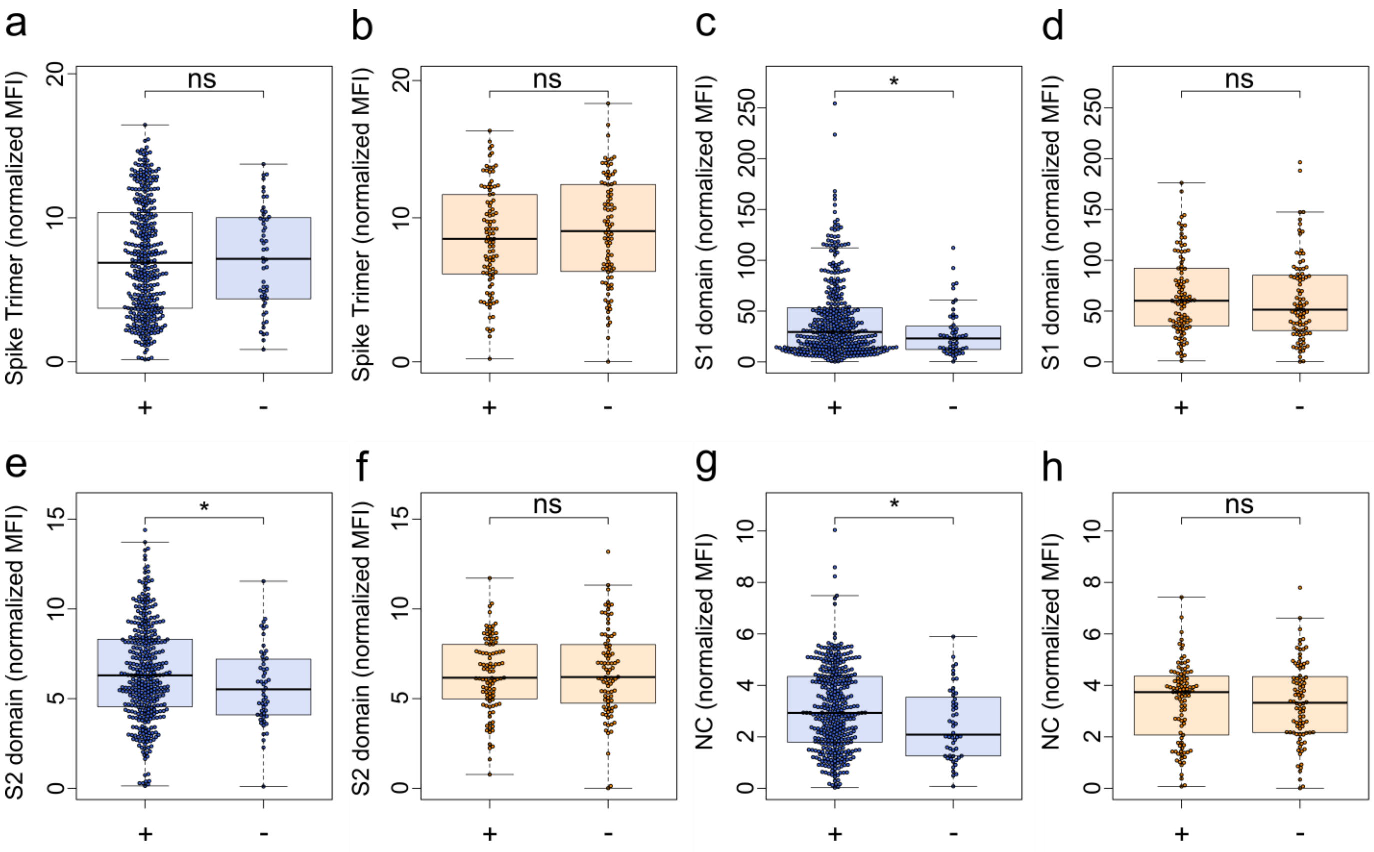

Figure S7 – **There is no difference in antibody response between asymptomatic and symptomatic infections in children**. Box and whisker plots with the box representing the median, 25th and 75th percentiles, while whiskers show the largest and smallest non-outlier values. Outliers were identified using upper/lower quartile ±1·5 times IQR. Statistical significance was calculated by Mann-Whitney-U (two-sided) with * indicating a p-value <0·05. and ns indicating a non-significant p-value >0·05. “+” indicates a symptomatic infection while “-“ indicates an asymptomatic infection. There were no significant differences between symptomatic and asymptomatic seropositive children (orange, n=185) in terms of antibody response for the spike trimer (b, p=0·43), S1 domain (d, p=0·34), S2 domain (f, p=0·87) or Nucleocapsid (NC) (h, p=0·78). Symptomatic and asymptomatic seropositive adults (blue, n=414) showed no significant difference for the spike trimer (a, p=0·94), although there were small significant differences in the S1 domain (c, p=0·03) and S2 domain (e, p=0·05) and NC (g, p=0·01). MFI – median fluorescence intensity.

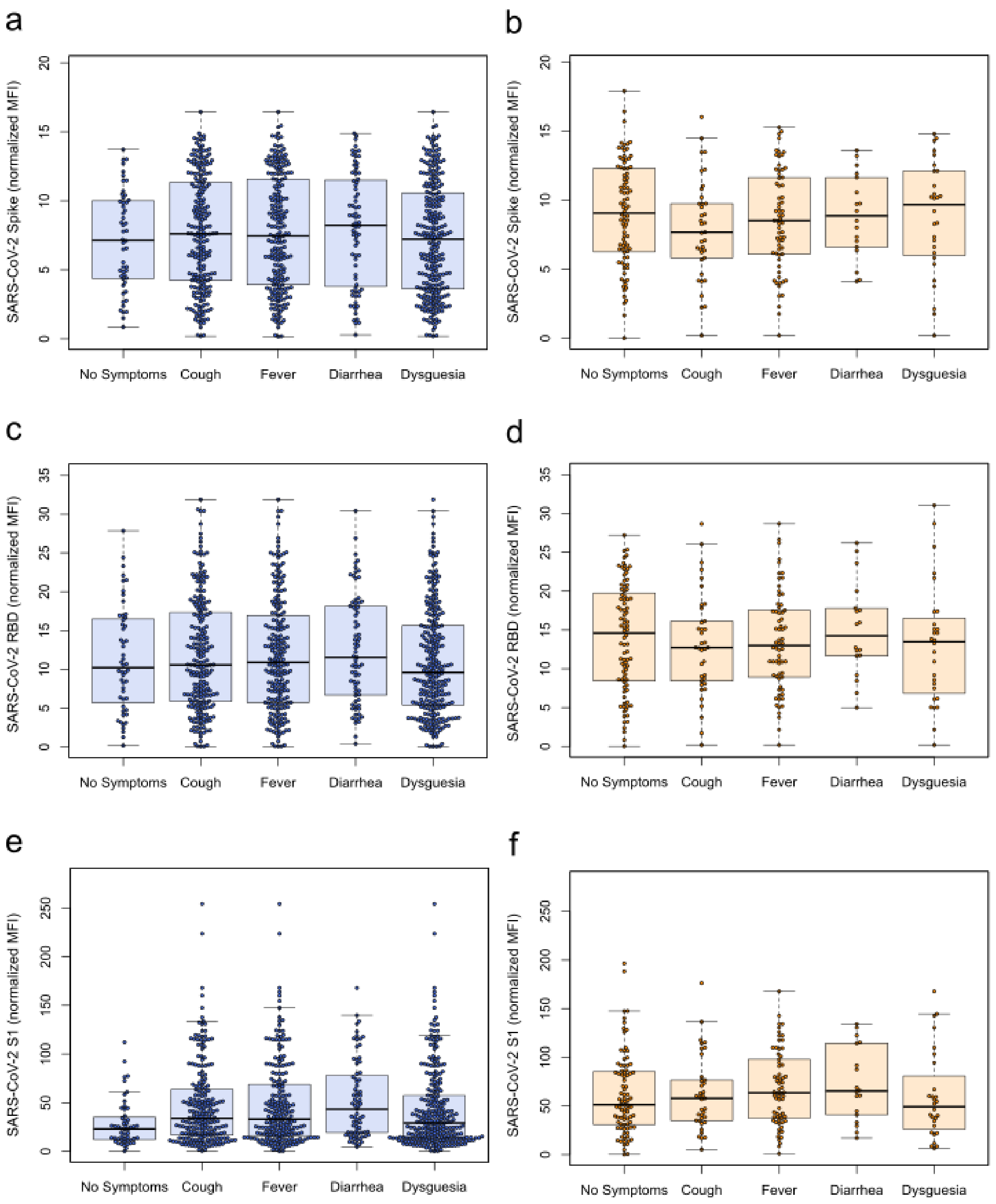

Figure S8 – **Symptoms do not moderate antibody response amongst seropositive individuals after mild COVID-19**. Box and whisker plots with the box representing the median, 25th and 75th percentiles, while whiskers show the largest and smallest non-outlier values. Outliers were identified using upper/lower quartile ±1·5 times IQR. Within the seropositive subgroups, neither adults (blue, a, c, e) nor children (orange, b, d, f) showed any difference in response based on presence of

symptoms for either the spike trimer (a and b), receptor binding domain (RBD) (c and d) or S1 domain (e and f) of SARS-CoV-2. Symptom group sizes: no symptoms – adults n=36, children n=83, cough – adults n=221, children n=37, fever – adults n=217, children n=66, diarrhea – adults n=75, children n=18, dysgeusia – adults n=266, children n=28. MFI – median fluorescence intensity.

a

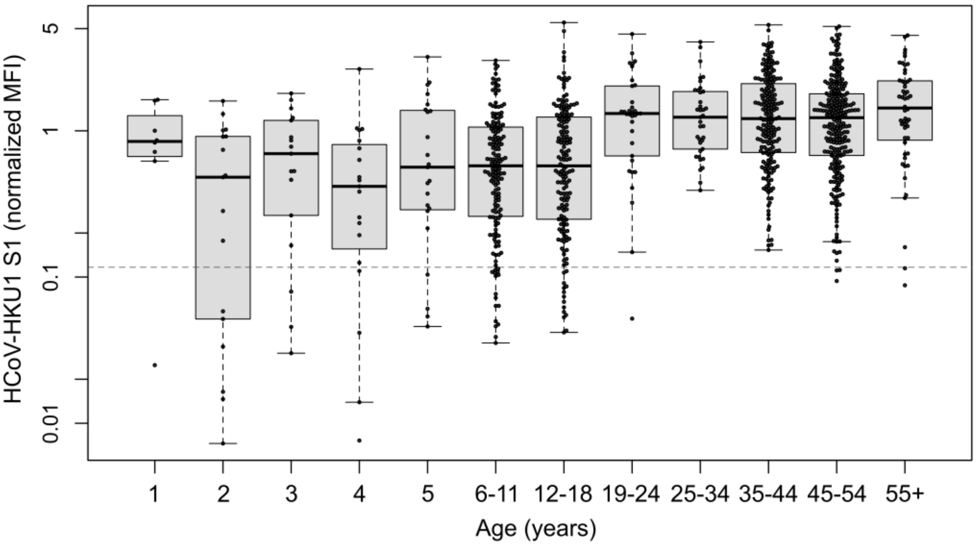
HCoV-HKU1 S1 (normalized MFI)
0.01
0.1
1
5
1
2
3
4
5
6-11
12-18
19-24
25-34
35-44
45-54
55+
Age (years)

b

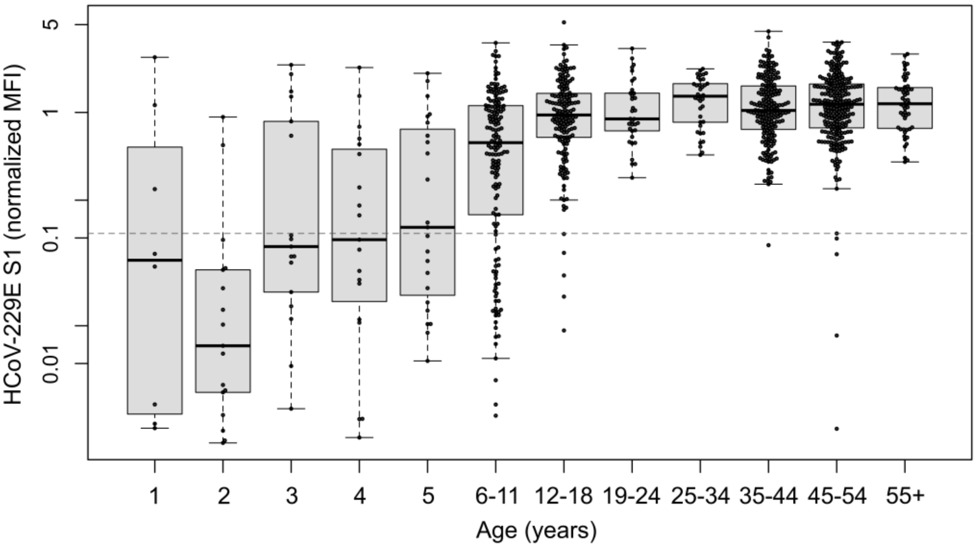
HCoV-229E S1 (normalized MFI)
0.01
0.1
1
5
1
2
3
4
5
6-11
12-18
19-24
25-34
35-44
45-54
55+
Age (years)

c

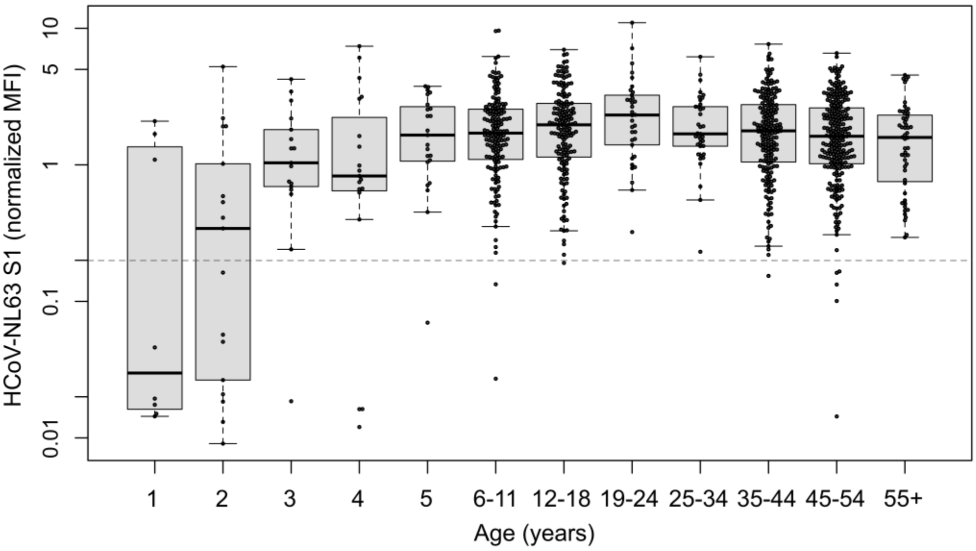
HCoV-NL63 S1 (normalized MFI)
0.01
0.1
1
5
10
1
2
3
4
5
6-11
12-18
19-24
25-34
35-44
45-54
55+
Age (years)

Figure S9 – **Initial HCoV infection often occurs during the first five years of life**. Box and whisker plots with the box representing the median, 25th and 75th percentiles, while whiskers show the largest and smallest non-outlier values. Outliers were identified using upper/lower quartile ±1·5 times IQR. Single ages from 1 to 5 are shown (1 – n=8, 2 – n=17, 3 – n=17, 4 – n=19, 5 – n=23) with age then grouped into: 6-11 (n=160), 12-18 (n=163), 19-24 (n=34), 25-34 (n=37), 35-44 (n=195), 45-54 (n=346) and over 55 years olds (n=52). For all HCoVs (a – HKU1, b – 229E, c – NL63), the majority of naïve samples are children. Dashed line indicates one-tenth of the mean response of all samples. All samples below the dashed line are considered to be naïve. HCoV – human endemic Coronavirus, MFI – median fluorescence intensity.

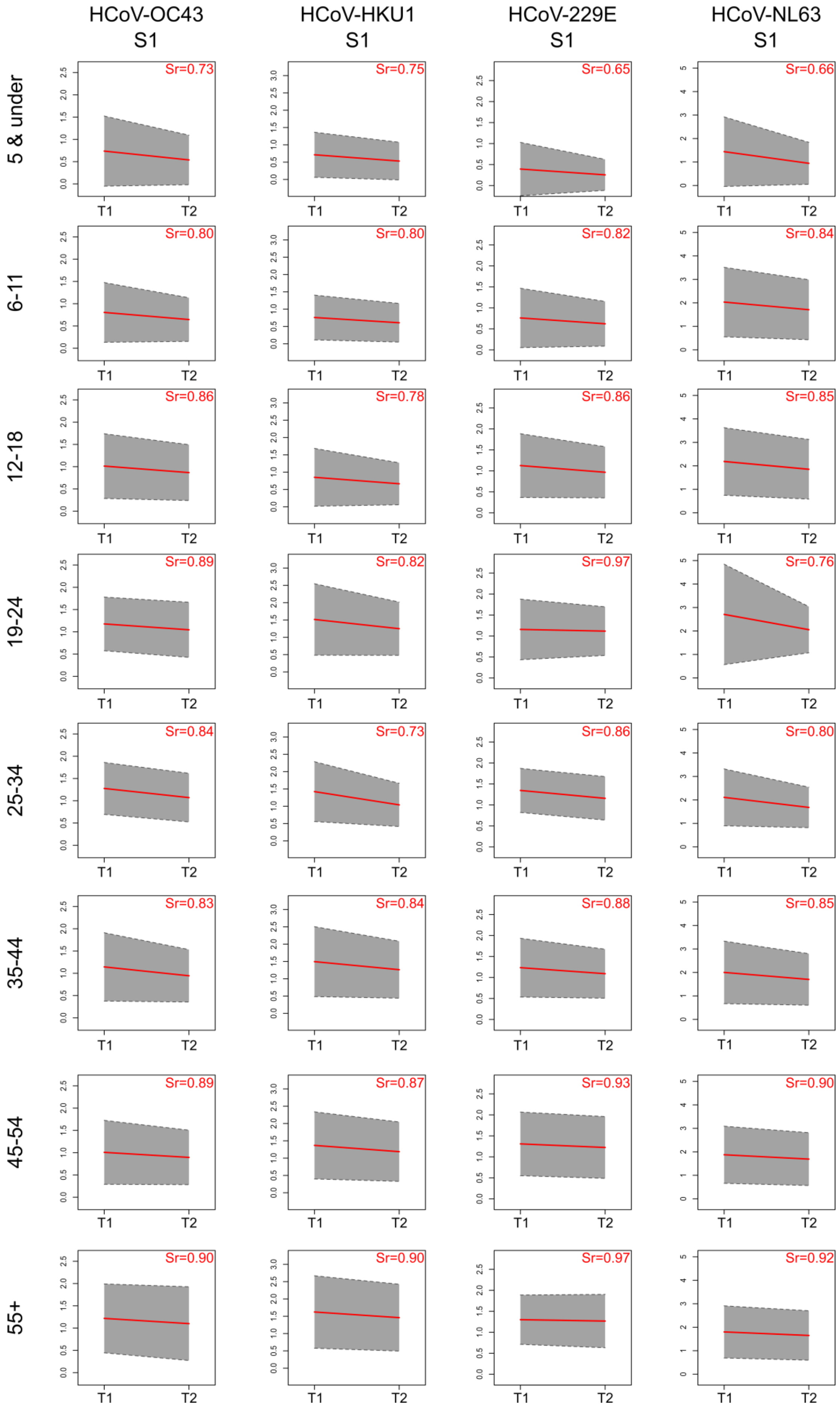
HCoV-OC43 S1
HCoV-HKU1 S1
HCoV-229E S1
HCoV-NL63 S1
5 & under
Sr=0.73
Sr=0.75
Sr=0.65
Sr=0.66
6-11
Sr=0.80
Sr=0.80
Sr=0.82
Sr=0.84
12-18
Sr=0.86
Sr=0.78
Sr=0.86
Sr=0.85
19-24
Sr=0.89
Sr=0.82
Sr=0.97
Sr=0.76
25-34
Sr=0.84
Sr=0.73
Sr=0.86
Sr=0.80
35-44
Sr=0.83
Sr=0.84
Sr=0.88
Sr=0.85
45-54
Sr=0.89
Sr=0.87
Sr=0.93
Sr=0.90
55+
Sr=0.90
Sr=0.90
Sr=0.97
Sr=0.92
T1
T2

Figure S10 – **Children serorevert faster for HCoVs than adults**. Longitudinal comparison of T1 and T2 samples using MULTICOV-AB to determine the rate of seroreversion. The S1 domain of HCoV-OC43, HCoV-HKU1, HCoV-229E and HCoV-NL63 are shown. Samples are separated into distinct age groups: five years old and under (n=84), 6-11 (n=160), 12-18 (n=162), 19-24 (n=32), 25-34 (n=36), 35-44 (n=182), 45-54 (n=228) and over 55 years old (n=48). Red lines indicate mean rate of decrease; grey boxes indicate ±1 standard deviation. Sr indicates the proportion of signal remaining, calculated as the ratio of the mean MFI at T2 compared to the mean MFI at T1. HCoV – human endemic Coronavirus, MFI – median fluorescence intensity.

a

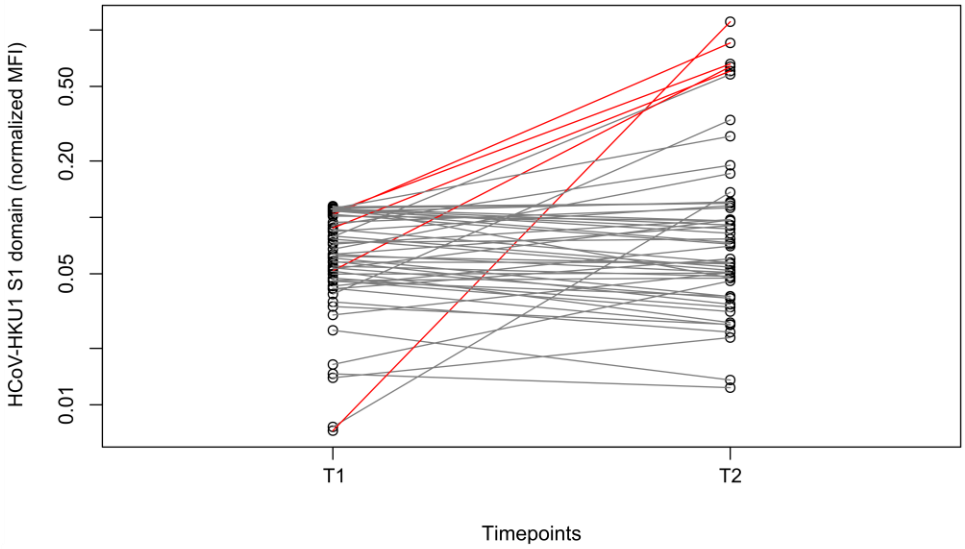
HCoV-HKU1 S1 domain (normalized MFI)
0.01
0.05
0.20
0.50
T1
T2
Timepoints

b

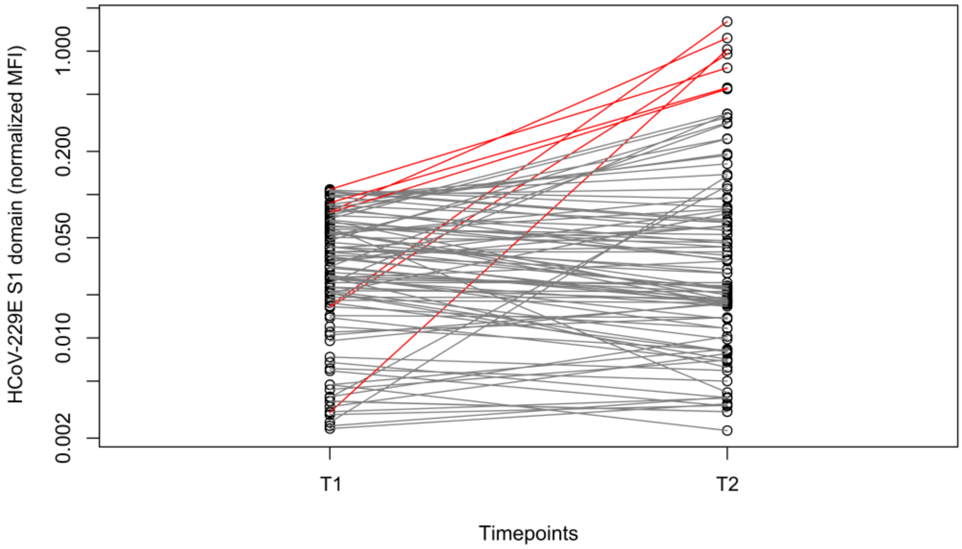
HCoV-229E S1 domain (normalized MFI)
0.002
0.010
0.050
0.200
1.000
T1
T2
Timepoints

c

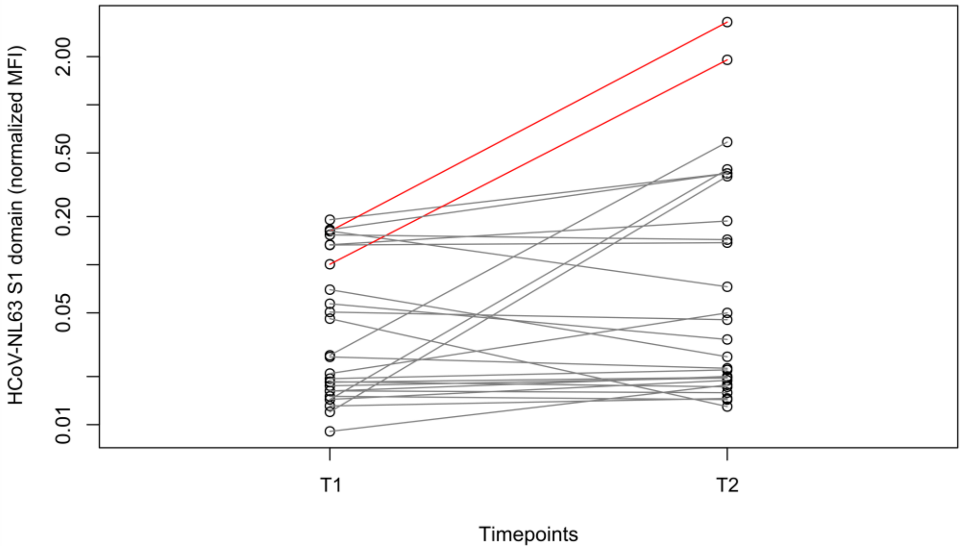
HCoV-NL63 S1 domain (normalized MFI)
0.01
0.05
0.20
0.50
2.00
T1
T2
Timepoints

Figure S11 – **Naïve samples are present within the study, although endemic coronavirus infections persisted during the SARS-CoV-2 pandemic**. Line graphs showing longitudinal antibody response from T1 to T2 for samples defined as naïve at T1. Individuals who remain naïve are shown in grey, individuals who seroconvert between T1 and by T2 are shown in red. Not all individuals who show increased HCoV antibody levels at T2 compared to T1 are considered to have been infected, as some remain within the negative range for the assay at T2. Normalized MFI is shown on a logscale for clarity. Although there is variation in the number of naïve samples between the different HCoVs (a – HKU1, b – 229E, c – NL63), new HCoV infections are seen across all HCoVs. HCoV – human endemic Coronavirus, MFI – median fluorescence intensity.

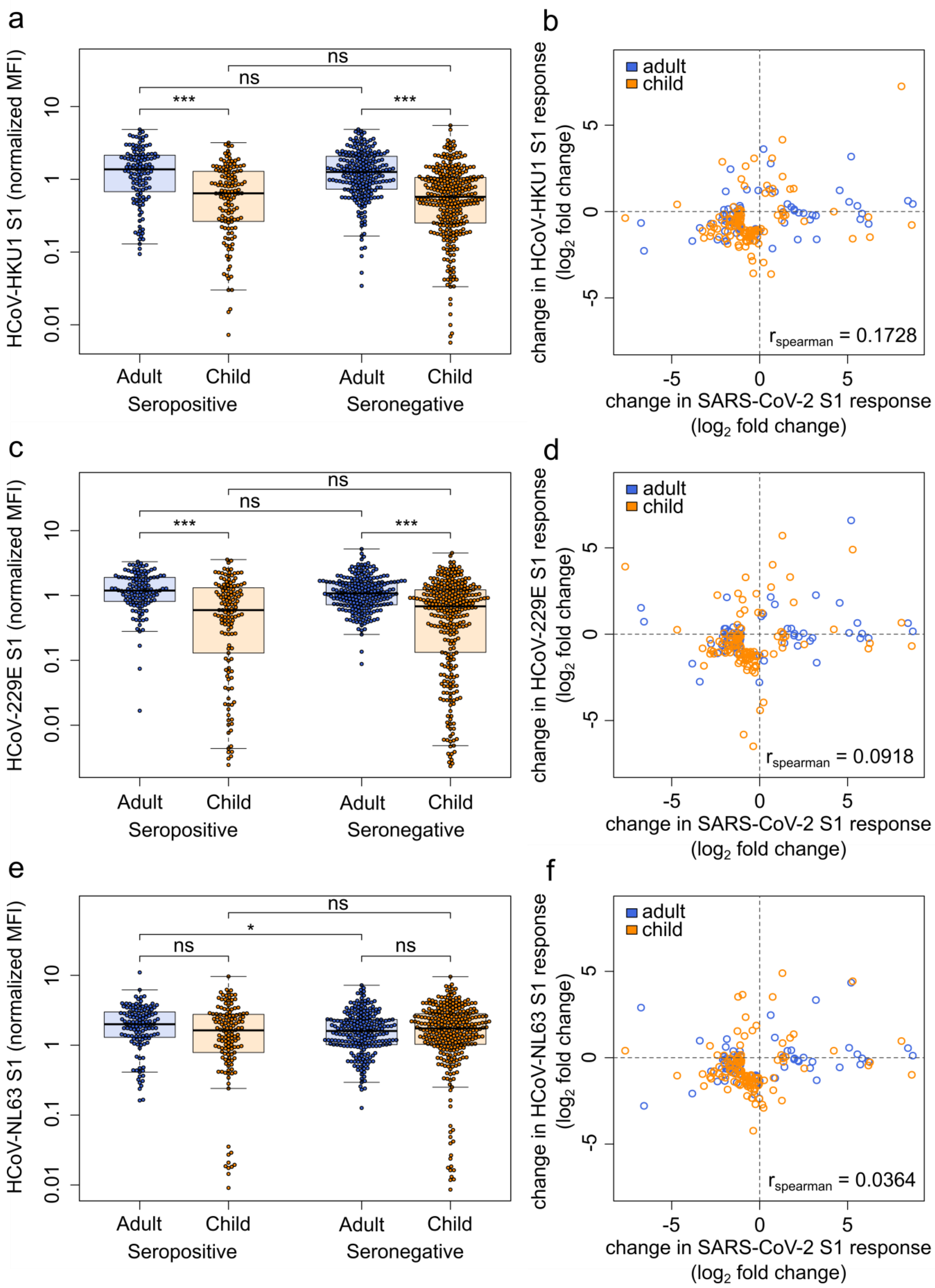
a
b
c
d
e
f
HCoV-HKU1 S1 (normalized MFI)
HCoV-229E S1 (normalized MFI)
HCoV-NL63 S1 (normalized MFI)
ns
***
*
Adult
Child
Seropositive
Seronegative
adult
child
change in HCoV-HKU1 S1 response (log2 fold change)
change in HCoV-229E S1 response (log2 fold change)
change in HCoV-NL63 S1 response (log2 fold change)
change in SARS-CoV-2 S1 response (log2 fold change)
rspearman = 0.1728
rspearman = 0.0918
rspearman = 0.0364

Figure S12 – **HCoVs offer no cross protection towards SARS-CoV-2, nor do they show a boost-back antibody response following SARS-CoV-2 infection**. Samples from households with a known index case were examined with MULTICOV-AB to determine whether the antibody response to endemic coronaviruses (HCoV) provides any protection against SARS-CoV-2. (a, c and e) Box and whisker plots demonstrating no significant difference between SARS-CoV-2 seropositive and seronegative adults (blue, n=440) or children (orange, n=436) in terms of HCoV-HKU1 (a, adult p=0·67, child p=0·47) or HCoV-229E (c, adult p=0·14, child p=0·99). For HCoV-NL63, there was a small significant difference for adults only (e, adults p=0·01, children p=0·35). Boxes represent the median, 25th and 75th percentiles, and whiskers show the largest and smallest non-outlier values. Outliers were identified using upper/lower quartile ± 1·5 times IQR. Statistical significance was calculated by Mann-Whitney-U (two-sided) with *** indicating a p-value <0·001, * indicating a p-value <0·05 and ns indicating a p-value >0·05 (b, d and f). When comparing paired samples longitudinally within the SARS-CoV-2 seropositive subgroup, there was no association between change in SARS-CoV-2 antibody level and change in HCoV antibody level for HCoV-HKU1 (b), HCoV-229E (d) or HCoV-NL63 (f) and in either adults (b - n=79, d – n=74, f – n=80) or children (b - n=117, d – n=114, f – n=118). Change in response is presented as log2-fold change from T1 to T2 and only samples with a log2-fold change > 1 or < -1 are shown. Spearman's rank was used to calculate ordinal associations between the changes in HCoV antibody level and change in SARS-CoV-2 antibody level. MFI – median fluorescence intensity.

Supplementary Tables

Table S1 – **Comparative seroprevalence between different assays used in the study**

Assay	Seropositive	Seronegative	Total	% of Seropositive
EuroImmun S1 IgG	991	1245	2236	44.3
Roche Elecsys N pan Ig	1149	1087	2236	51·4
Siemens RBD IgG	1067	1169	2236	47·7
MULTICOV S and RBD IgG	1142	1094	2236	51·1

Only samples that were measured with all four assays are considered. For each assay, the manufacturer or name of the assay is stated, as well as the target antigen and Ig-isotype detected. N – Nucleocapsid, RBD – receptor binding domain, S – spike protein.

Table S2 **– Symptom frequency and diagnostic performance in children under 18**

		Age group						All children	
		Under 5		6 to 11		12 to 18			
Symptom	Present or Absent	n (%)	Seropositive (PPV, 95% CI)	n (%)	Seropositive (PPV, 95% CI)	n (%)	Seropositive (PPV, 95% CI)	n (%)	Seropositive (PPV, 95% CI)
Fever	Present	37 (28·03)	17 (0·46, 0·33-0·59)	46 (21·70)	28 (0·61, 0·48-0·72)	28 (13·40)	21 (0·75, 0·57-0·87)	111 (20·07)	66 (0·59, 0·51-0·67)
	Absent	95 (71·97)	23 (0·24, 0·19-0·30)	166 (78·30)	45 (0·27, 0·24-0·31)	181 (86·60)	53 (0·29, 0·27-0·32)	442 (79·93)	121 (0·27, 0·25-0·29)
Cough	Present	28 (21·21)	9 (0·32, 0·19-0·48)	43 (20·28)	14 (0·33, 0·21-0·46)	28 (13·40)	14 (0·50, 0·34-0·66)	99 (17·90)	37 (0·37, 0·29-0·46)
	Absent	104 (78·79)	31 (0·30, 0·25-0·35)	169 (79·72)	59 (0·35, 0·31-0·38)	181 (86·60)	60 (0·33, 0·30-0·36)	454 (82·10)	150 (0·33, 0·31-0·35)
Diarrhea	Present	8 (6·06)	3 (0·38, 0·13-0·71)	17 (8·02)	11 (0·65, 0·41-0·83)	8 (3·83)	4 (0·50, 0·20-0·80)	33 (5·97)	18 (0·55, 0·38-0·70)
	Absent	124 (93·94)	37 (0·30, 0·27-0·32)	195 (91·98)	62 (0·32, 0·30-0·34)	201 (96·17)	70 (0·35, 0·33-0·36)	520 (94·03)	169 (0·33, 0·31-0·34)
Dysguesia	Present	1 (0·76)	1 (1·00, n/a)	7 (3·30)	6 (0·86, 0·42-0·98)	24 (11·48)	21 (0·88, 0·68-0·96)	32 (5·79)	28 (0·88, 0·71-0·95)
	Absent	131 (99·24)	39 (0·30, 0·29-0·30)	205 (69·70)	67 (0·33, 0·32-0·34)	185 (88·52)	53 (0·29, 0·27-0·31)	521 (94·21)	159 (0·31, 0·30-0·31)

The frequency of each symptom within the study population, shown number of individuals (n, also as %) either with (present) or without (absent) this symptom, and the number of individuals (n, also as %) within these groups who were seropositive for SARS-CoV-

2. Children are split into three groups: under 5-year olds (n=132), 6- to 11-year olds (n=212) and 12- to 18-year olds (n=209). Positive Predictive Value (PPV) for seropositivity in the presence or absence of each symptom, 95% Confidence Intervals (CI) are standard logit confidence intervals[1].

Table S3 – **List of antigens used in MULTICOV-AB**

Disease	Antigen	Manufacturer	Cat. No.
SARS-CoV-2	Spike Trimer	NMI	-
SARS-CoV-2	RBD	NMI	-
SARS-CoV-2	S1 domain	NMI	-
SARS-CoV-2	S2 domain	Sino	40590
SARS-CoV-2	Nucleocapsid	Aalto	6404-b
SARS-CoV-2	Nucleocapsid N-terminal domain	NMI	-
SARS-CoV-2	RBD alpha variant	NMI	-
SARS-CoV-2	RBD beta variant	NMI	-
HCoV-OC43	S1 domain	NMI	-
HCoV-OC43	Nucleocapsid	NMI	-
HCoV-OC43	Nucleocapsid N-terminal domain	NMI	-
HCoV-HKU1	S1 domain	NMI	-
HCoV-HKU1	Nucleocapsid	NMI	-
HCoV-HKU1	Nucleocapsid N-terminal domain	NMI	-
HCoV-NL63	S1 domain	NMI	-
HCoV-NL63	Nucleocapsid	NMI	-
HCoV-NL63	Nucleocapsid N-terminal domain	NMI	-
HCoV-229E	S1 domain	NMI	-
HCoV-229E	Nucleocapsid	NMI	-
HCoV-229E	Nucleocapsid N-terminal domain	NMI	-

List of antigens included in MULTICOV-AB in this study, including information about their manufacturer, and if available, their category number. Full information on the NMI produced antigens can be found at[2,3].

Supplementary References

1. Mercaldo N.D, Lau K.F and Zhou X.H. Confidence intervals for predictive values with an emphasis to case-control studies. Statist Med 2007;26:2170-2183.
2. Becker M, Dulovic A, Junker D, et al. Immune response to SARS-CoV-2 variants of concern in vaccinated individuals. Nat Commun 2021;12(1):3109.
3. Becker M, Strengert M, Junker D, et al. Exploring beyond clinical routine SARS-CoV-2 serology using MultiCoV-Ab to evaluate endemic coronavirus cross-reactivity. Nat Commun 2021;12(1):1152.
