# Supplementary material for: Robust and durable serological response following pediatric SARS-CoV-2 infection
Source: Nat Commun. 2022 Jan 10;13:128. doi: 10.1038/s41467-021-27595-9 (PMC8748910; doi:10.1038/s41467-021-27595-9)
Supplement: Supplementary file 3 — Reporting Summary [file 41467_2021_27595_MOESM3_ESM.pdf]

nature portfolio

Corresponding author(s): Roland Elling

Last updated by author(s): Nov 3, 2021

Reporting Summary

Nature Portfolio wishes to improve the reproducibility of the work that we publish. This form provides structure for consistency and transparency in reporting. For further information on Nature Portfolio policies, see our Editorial Policies and the Editorial Policy Checklist.

Statistics

For all statistical analyses, confirm that the following items are present in the figure legend, table legend, main text, or Methods section.

n/a	Confirmed	
☐	☒	The exact sample size (*n*) for each experimental group/condition, given as a discrete number and unit of measurement
☐	☒	A statement on whether measurements were taken from distinct samples or whether the same sample was measured repeatedly
☐	☒	The statistical test(s) used AND whether they are one- or two-sided<br>*Only common tests should be described solely by name; describe more complex techniques in the Methods section.*
☐	☒	A description of all covariates tested
☒	☐	A description of any assumptions or corrections, such as tests of normality and adjustment for multiple comparisons
☐	☒	A full description of the statistical parameters including central tendency (e.g. means) or other basic estimates (e.g. regression coefficient) AND variation (e.g. standard deviation) or associated estimates of uncertainty (e.g. confidence intervals)
☐	☒	For null hypothesis testing, the test statistic (e.g. *F*, *t*, *r*) with confidence intervals, effect sizes, degrees of freedom and *P* value noted<br>*Give P values as exact values whenever suitable.*
☒	☐	For Bayesian analysis, information on the choice of priors and Markov chain Monte Carlo settings
☒	☐	For hierarchical and complex designs, identification of the appropriate level for tests and full reporting of outcomes
☐	☒	Estimates of effect sizes (e.g. Cohen's *d*, Pearson's *r*), indicating how they were calculated

Our web collection on statistics for biologists contains articles on many of the points above.

Software and code

Policy information about availability of computer code

Data collection	Data was collected using the following instruments and software:<br>Luminex FLEXMAP 3D instrument with xPONENT Software 4.3<br>Orion II Microplate Luminometer with Simplicity Software v4.2<br>Cobas e411 or e811 Platform<br>Advia Centaur XPT Platform<br>POLARstar Omega Plate Reader<br>BEP2000 Advance Siemens Healthineers
Data analysis	Pre-processing of data such as matching metadata and sample IDs was performed in Excel 2016. Initial data collection was done using Microsoft Excel and Access. Formal data analysis was performed on RStudio (Version 1.2.5001, running R 3.6.1) with the following additional packages: "RColorBrewer (version 1.1-2)", "beeswarm (version 0.2.3)", "gplots (version 3.0.1.1)", "Venn Diagram (version 1.6.20)", all of which were used solely for data depiction and not statistical analysis. Figures were generated in RStudio and then edited for clarity in Inkscape (Inkscape 0.92.4). Only samples from time point 1 were used for all non-longitudinal analyses. For longitudinal analyses, only those participants for whom both Tl and T2 samples were available were included and all participants who were vaccinated prior to were excluded. For analysis of potential cross-protection though endemic coronaviruses, only households with a known index case were used and the index case itself was excluded. Statistical analyses performed are described in the figure legends. For comparison of signal distribution between sample groups, two-way Mann-Whitney-U tests were performed using the "wilcox.test" function from R's "stats(base library in v.3.6.1)" library. For correlation analysis, Spearman's rank was calculated using the "cor" function from R's "stats" library. p-values <0.01 were considered to be significant. GraphPad Prism version 9.1.1 was used to process Delta neutralization assay data. Due to the lack of availability of the underlying data, we are currently not making the analysis code publically available, as it could not be used for reconstructing the analysis.

For manuscripts utilizing custom algorithms or software that are central to the research but not yet described in published literature, software must be made available to editors and reviewers. We strongly encourage code deposition in a community repository (e.g. GitHub). See the Nature Portfolio guidelines for submitting code & software for further information.

Data

Policy information about availability of data

All manuscripts must include a data availability statement. This statement should provide the following information, where applicable:

- Accession codes, unique identifiers, or web links for publicly available datasets
- A description of any restrictions on data availability
- For clinical datasets or third party data, please ensure that the statement adheres to our policy

Individual participant data, including data dictionaries cannot be made publically available as per the terms of the Ethics Agreement of this study. For child data, parental consent has not been obtained for data sharing.

Field-specific reporting

Please select the one below that is the best fit for your research. If you are not sure, read the appropriate sections before making your selection.

[x] Life sciences [ ] Behavioural & social sciences [ ] Ecological, evolutionary & environmental sciences

For a reference copy of the document with all sections, see nature.com/documents/nr-reporting-summary-flat.pdf

Life sciences study design

All studies must disclose on these points even when the disclosure is negative.

Sample size	Sample size was based on maximal available samples who had all relevant metadata collected and had been analysed with all four antibody assays. For some assays such as Neutralization, a subset of samples was used which is indicated in the corresponding section of the manuscript and figure legend.
Data exclusions	No data was excluded from analysis.
Replication	Performance of MULTICOV-AB was verified with quality control samples processed on every plate. As all plates passed quality control, these experiments were not replicated. All measurements made using commercially available antibody assays were performed with the appropriate calibrator and control samples and so were not replicated. As samples were analysed with multiple serological assays and seropositivity was determined on the basis on being positive in multiple independent assays, it was deemed acceptable to measure each sample once only with each assay. The Surrogate SARS-CoV-2 Neutralization Test from Genscript was performed once with the relevant controls as per the manufacturers instructions and so was not repeated. Neutralization assay with pseudoviruses containing SARS-CoV-2-Spike were performed in technical duplicates with commercially available antibodies (Bamlanivimab, Casivirimab, Imdevimab) analyzed at the same time to verify assay performance. WERE ALL REPLICATION ATTEMPTS SUCCESSFUL FOR NEUTRALIZATION?
Randomization	Serological antibody assays were measured randomly at one single timepoint with no seperation or identification based upon clinical covariables used. Samples were delivered to central laboratories, were they were processed in the order in which they arrived. Laboratory staff were blind to all metadata including sample origin. Neutralization assays used a subset of the study population which was randomly selected.
Blinding	Serological analysis was performed blinded for all clinical covariables for all antibody assays. Investigators were blinded towards all group allocation, the allocation of samples into subgroups (e.g. only those measured for neutralization) until after all experiments had been completed. The results of all commercial assays and pseudovirus neutralisation assay were only combined once all had been completed. No metadata or identifiers were available to either the experimental labs or the data analysis group until after all experiments had been completed.

Reporting for specific materials, systems and methods

We require information from authors about some types of materials, experimental systems and methods used in many studies. Here, indicate whether each material, system or method listed is relevant to your study. If you are not sure if a list item applies to your research, read the appropriate section before selecting a response.

Materials & experimental systems

n/a	Involved in the study	
[ ]	[x]	Antibodies
[ ]	[x]	Eukaryotic cell lines
[x]	[ ]	Palaeontology and archaeology
[x]	[ ]	Animals and other organisms
[ ]	[x]	Human research participants
[ ]	[x]	Clinical data
[x]	[ ]	Dual use research of concern

Methods

n/a	Involved in the study	
[x]	[ ]	ChIP-seq
[x]	[ ]	Flow cytometry
[x]	[ ]	MRI-based neuroimaging

Antibodies

Antibodies used	Antibodies bound to microspheres were detected with R-phycoerythrin labeled goat-anti-human IgG or IgA antibodies:<br>goat-anti-human-IgG-RPE (BIOZOL, Cat# 109-116-098, Lot#149288)<br>goat-anti-human-IgA-RPE (BIOZOL, Cat# 109-115-011, Lot#149254)<br>Anti-V-VSG antibody was used in the pseudovirus neutralization experiments - I1-Hybridoma (ATCC CRL-2700'M) NEED MANUFACTURER, CAT AND LOT NUMBER FOR THIS.
Validation	Both RPE-labelled detection antibodies used in the developed assays are widely used in assays based on xMAP Technology and are Immunoglobulin subtype specific as stated on the manufacturers website. Both RPE-antibodies are human specific with no cross-reactivity against bovine, horse or mouse (per manufacturer). We have further cross-validated the antibodies by measuring backgound signals when incubating with microspheres coated with human IgA or human IgG and did not find notable immunoglobulin-species cross-reactivity. I1-Hybridoma was used to block residual VSV-G containing particles in pseudovirus stocks and validated to not interfere with infection of Spike-containing particles.

Eukaryotic cell lines

Policy information about cell lines

Cell line source(s)	VeroE6 (ATCC CRL-1586)<br>HEK-293T PLEASE FILL OUT THE MISSING INFORMATION FOR THE HEK CELLS IN ALL OF THIS SECTION
Authentication	VeroE6 were purchased from ATCC and not further authenticated.<br>HEK-293T<br>PLEASE ALSO STATE THE METHODS USED BY THE MANUFACTURER FOR AUTHENTICATION IF YOU DID NOT AUTHENTICATE e.g. STR profiling, karyotyping etc
Mycoplasma contamination	VeroE6 were regularly tested for mycoplasma contamination and tested negative.<br>HEK-293T
Commonly misidentified lines<br>(See ICLAC register)	None

Human research participants

Policy information about studies involving human research participants

Population characteristics	We did a secondary analysis of a non-interventional, prospective observational national multi-center cohort study, including 548 children and 717 adults within 328 households with at least one SARS-CoV-2 RT-PCR-confirmed or one seropositive, symptomatic individual. Serum samples were collected at two seperate timepoints, median 109 days (IQR 67-122) after earliest household symptom onset and 340 days (IQR 322-356). Participants were asked to fill in questionnaire at both ti mepoi nts. The following inclusion criteria were applied: Participants must have been children aged 1 to 18 years, parents or other adults living in the same household with the investigated children (no gender bias or age limit), be resident in the state of Baden-Wurttemberg and provide written consent to the study. The following exclusion criteria were applied: severe congenital disease, congential or acquired immunodeficiencies and insufficient comprehension of German.
Recruitment	Participants were recruited during the first wave of the pandemic in Germany (May to August 2020) via local authorities and an in-hospital database of household with at least one laboratory-confirmed SARS-CoV-2 infection. Please outline any potential self-selection bias or other biases that may be present and how these are likely to impact results???
Ethics oversight	Ethics approval was obtained from the respective Medical Faculties' independent ethics committees (University of Freiburg: 256/20_201553; University of Tubingen: 293/2020BO2; University of Ulm: 152/20). Written informed consent was obtained from adult participants and from parents or legal guardians on behalf of their children at both sampling time points. Children's preferences on whether or not to provide a blood sample were respected throughout.

Note that full information on the approval of the study protocol must also be provided in the manuscript.

Clinical data

Policy information about clinical studies

All manuscripts should comply with the ICMJEguidelines for publication of clinical research and a completedCONSORT checklist must be included with all submissions.

Clinical trial registration	This study was registered at the German Clinical Trials Register (DRKS), study ID 00021521
Study protocol	The full study protocol is available from https://www.drks.de/drks_web/navigate.do?navigationId=trial.HTML&TRIAL_ID=DRKS00021521
Data collection	hildren and adults within eligible households completed a questionnaire containing demographic information (date of birth, gender, height, weight, smoking), the presence of symptoms (fever, cough, dysgeusia or diarrhea) in plausible temporal association (max. 2 weeks prior or later) with the onset of the SARS-CoV-2 infection within the household or around the time of a positive SARS-CoV-2 RT-PCR, and symptom duration. They additionally provided serum samples for immunological analysis at time point 1 and at follow-up time point 2 after the SARS-CoV-2 infection within the household. Data on vaccination and potential re-infection within the

household were collected at time point 2. We investigated all invited households with at least one child to avoid selection bias. Questionnaires were checked for missing or inadequate data and inconsistencies; where possible, these points were clarified retrospectively with the families. To predetermine the sample size, we used a one-factor variance analysis design. Assuming 1.5 children per household participating in the study and 3 different age ranges, we aimed at a sample size of approximately 200 households to reveal small effect sizes of about 0.1 at a significance level of 5% and a test strength of 80%. Please provide details on the settings and places where the data were collected as well as the periods of time for recruitment and data collection??

Outcomes

Primary outcome measures: Seroprevalence and titers of SARS-CoV-2 antibodies in children and adults from the same household with at least one index case with proven SARS-CoV-2 infection (positive RT-PCR test or symptomatic and later serologically proven infection) with or without COVID-19 disease. Secondary outcome measures:

a) Characterize the humoral immune response to SARS-CoV-2 in children and adults by 3 commercial SARS-CoV-2 antibody assays and a bead-based multiplex immunoassay, including Variants of Concern and endemic human coronaviruses.

b) Assess a potential relationship between antibody titers and symptoms in the setting of acute infection.

c) Assess age-related endemic human coronavirus seroprevalence and investigate potential cross-protection of HCoV antibodies against SARS-CoV-2.

d) Assess persistence and titers of detectable SARS-CoV-2 antibodies over a period of 11-12 months in children and adults.

e) Assess neutralization capacity against wild type SARS-CoV-2 and the delta Variant of Concern

Please describe how the primary and secondary outcomes were pre-defined and how these measures were assessed??
